# Large language models show distinct multidimensional performance profiles and variable reproducibility in consensus‐based bone stress injury

**DOI:** 10.1002/jeo2.70939

**Published:** 2026-10-07

**Authors:** Silvia Gandini, Jakob Lindskog, Yinan Yu, Felix Öttl, Richard Willy, Paolo Emilio Adami, Francesco Della Villa, Stefano Di Paolo, Fabio Bonsanto, Stefano Mazzoni, Francesco Feletti, Kristian Samuelsson, Eric Hamrin Senorski

**Affiliations:** ^1^ Milan Lab Research Department AC Milan S.p.A Milan Italy; ^2^ Department of Biomedical Sciences University of Padova Padua Italy; ^3^ Unit of Physiotherapy, Department of Health and Rehabilitation, Institute of Neuroscience and Physiology, Sahlgrenska Academy University of Gothenburg Gothenburg Sweden; ^4^ Sahlgrenska Sports Medicine Center, Sahlgrenska Academy Gothenburg Sweden; ^5^ Department of Computer Science and Engineering Chalmers University of Technology and University of Gothenburg Gothenburg Sweden; ^6^ Department of Orthopedic Surgery, Balgrist University Hospital University of Zürich Zurich Switzerland; ^7^ School of Health & Rehabilitation Sciences The Ohio State University Columbus Ohio USA; ^8^ Sports Medicine Research Institute, The Ohio State University Wexner Medical Center The Ohio State University Columbus Ohio USA; ^9^ Health and Science Department World Athletics Monaco; ^10^ Physiology and Pharmacology Department “V. Erspamer” Sapienza University of Rome Rome Italy; ^11^ Education and Research Department, Isokinetic Medical Group FIFA Medical Centre of Excellence Bologna Italy; ^12^ Department of Translational Medicine and for Romagna University of Ferrara Ferrara Italy; ^13^ Radiology Unit of Ravenna, S. Maria delle Croci Hospital, AUSL Romagna Ravenna Italy; ^14^ EXTREMESPORTMED, International Society of Medicine for Outdoor Physical Activity and Extreme Sports Imola Bologna Italy; ^15^ Department of Orthopaedics, Institute of Clinical Sciences, Sahlgrenska Academy University of Gothenburg Gothenburg Sweden; ^16^ Department of Orthopaedics Sahlgrenska University Hospital Mölndal Sweden; ^17^ Swedish Olympic Committee Stockholm Sweden

**Keywords:** artificial intelligence, bone stress injuries, Delphi consensus, large language models, sports medicine

## Abstract

**Purpose:**

This study evaluated five publicly available large language model‐based systems using a multidimensional, expert‐based assessment of consensus‐based clinical questions on bone stress injuries.

**Methods:**

Ten standardised questions derived from a 2025 international Delphi consensus on bone stress injuries were submitted to ChatGPT‐5, Perplexity AI, Claude 4, Google Gemini 2.5 and Microsoft Copilot. Responses were independently rated by clinical experts using a multidimensional evaluation framework covering accuracy, comprehensiveness, understanding, reasoning, clarity, potential harm and trust, each rated on a 5‐point Likert scale. Median scores were calculated for each question and model, and comparisons were performed using Kruskal–Wallis tests followed by Dunn's post hoc tests with Bonferroni correction. Interrater agreement was assessed using the weighted Gwet's agreement coefficient. Reproducibility was assessed by repeatedly submitting nine questions representing no, partial, and full expert consensus to each model, with response consistency scored from 1 to 3.

**Results:**

Significant differences between models were observed for comprehensiveness, reasoning, clarity and trust (all *p* < 0.001), with no differences in accuracy or harm. ChatGPT‐5 and Claude 4 achieved significantly higher comprehensiveness scores, while ChatGPT‐5 and Google Gemini 2.5 had significantly higher reasoning scores than Microsoft Copilot. Claude 4 demonstrated significantly lower clarity than all other models. ChatGPT‐5 achieved significantly higher trust scores and showed the highest mean reproducibility score, followed by Perplexity AI and Gemini 2.5; Microsoft Copilot showed the lowest reproducibility. Interrater agreement ranged from fair to almost perfect, with substantial overall agreement.

**Conclusions:**

Publicly available large language model‐based systems demonstrated moderate‐to‐high performance in addressing expert‐derived, consensus‐based questions on bone stress injuries, suggesting potential to support the communication of consensus‐based knowledge. However, performance varied across evaluation dimensions and reproducibility, indicating that large language models may support, but should not replace, primary consensus documents or expert clinical judgement.

**Level of Evidence:**

N/A.

## Graphical abstract

Graphical illustration of study.

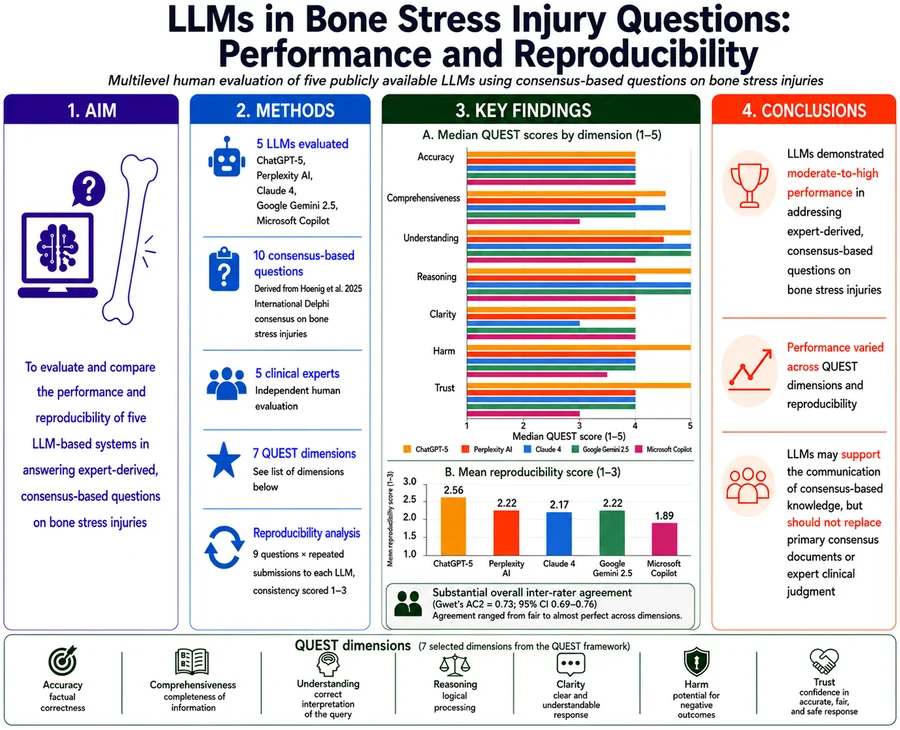

**Abbreviations**
AC2: Gwet's agreement coefficient
AI: artificial intelligence
BSI: bone stress injury
BSIs: bone stress injuries
CI: confidence interval
IQR: interquartile range
LLM: large language model
LLMs: large language models
QUEST: quality, understanding and reasoning, expression style, safety and harm and trust and confidence
SRQR: standards for reporting qualitative research

## INTRODUCTION

Since the early 2020s, artificial intelligence (AI) has seen a rapid rise in popularity, particularly through the development of large language models (LLMs). Chatbot tools based on LLMs are increasingly used in healthcare for patient communication, guideline synthesis and decision support [3, 36] with generally accurate, although variable performance depending on clinical complexity [8, 9]. Previous studies in orthopaedics have evaluated LLM outputs in terms of correctness, completeness and adaptability across different target audiences, which highlights their potential as supplementary tools for medical communication and education [17, 18]. In orthopaedics and sports medicine, AI has been used to analyze large datasets, predict surgical outcomes and explore diagnostic algorithms [13, 14, 24, 43]. However, evidence on the ability of LLMs to accurately answer expert‐level consensus questions remains limited. Moreover, beyond accuracy, the reproducibility and stability of AI‐generated outputs represent important considerations in the evaluation of reliability and potential clinical use of LLM‐based systems [34].

To date, only limited work has directly evaluated LLM responses against expert‐derived Delphi consensus statements, such as the recent study by Vivekanantha et al. [41] that focused on patellar instability and showed higher accuracy ratings for ChatGPT and Claude compared with other LLMs. Several studies in orthopaedics (e.g., anterior cruciate ligament reconstruction, ulnar collateral ligament injury, hip arthroscopy) report generally satisfactory responses, although limitations such as incomplete or inconsistent information persist [2, 15, 22].

Bone stress fractures and bone stress reactions, collectively referred to as bone stress injuries (BSIs), represent a spectrum of overuse conditions that result from repetitive mechanical loading with insufficient recovery [6, 10]. While uncommon in the general population, BSIs are prevalent in high‐impact activities such as running [19], jumping [25] and dancing [16], and are influenced by multifactorial risk factors, including low energy availability, biomechanics and training load errors [31, 38, 39]. Due to their clinical complexity and reliance on integrating pathophysiological, imaging and functional information, BSIs represent a suitable model to differentiate between superficial knowledge retrieval and higher‐level clinical reasoning in LLMs [23].

Given the diversity of clinical approaches and the evolving literature, consensus‐based recommendations provide a structured reference for evaluating the alignment of LLM outputs with current expert knowledge. In this context, a multidimensional and human‐centred evaluation is essential to capture clinically relevant aspects of LLM performance beyond correctness alone. Therefore, this study adopted a multi‐level human evaluation approach based on the QUEST framework [33, 40], which enables structured assessment across multiple clinically relevant domains. The aim is to achieve a more comprehensive characterisation of LLM outputs, which reflects not only what information is provided but also how it is interpreted, structured and communicated.

This study evaluates responses generated by five publicly available LLM‐based systems (ChatGPT‐5, Perplexity AI, Claude 4, Microsoft Copilot and Google Gemini 2.5) to expert‐formulated questions derived from a Delphi consensus study on BSIs [12], and compares their performance based on reviewer‐assigned scores to explore their potential role in supporting evidence‐based practice in sports medicine. In addition, the study examines the reproducibility of model outputs across repeated submissions to assess their stability within a consensus‐based clinical context.

## MATERIALS AND METHODS

### Study design

The study was designed as a comparative analysis of LLM responses to consensus‐based clinical questions derived from an international Delphi study on BSIs [12]. The methodology consisted of two complementary components: (1) an expert‐based multidimensional evaluation of model responses to 10 selected questions, and (2) a reproducibility analysis assessing response stability across repeated submissions using nine additional questions representing different levels of expert consensus.

### Phase 1: Expert‐based human evaluation

The ten BSI questions were purposively selected to represent the main clinical domains covered by the Delphi consensus and included statements that had reached high levels of expert agreement. The questions focused on clinical topics including pathophysiology, diagnosis, terminology and classification systems; risk factors, screening and prevention; and management and return to sport. The selected items included statements with full consensus and statements classified as agreement despite some disagreement or abstention. The Delphi‐derived questions used for the expert‐based human evaluation are summarised in Table 1. Full Delphi‐derived questions submitted to the LLMs are provided in Supporting Information: Table S1, Online Resource 1.

**Table 1:** Delphi‐derived questions based on the 2025 International consensus on bone stress injuries [12], used for the human evaluation of large language model responses.

Question number	Clinical topic	Consensus level (%)	Delphi statements
1	Pathophysiology	≥96.4	1–4
2	Terminology and classification	≥81.5	5–8, 13–14
3	Clinical suspicion	79.3–96.6	15
4	Diagnostic assessment	≥82.1	17–21, 24, 26, 31
5	Risk factors	88.0–100	23
6	Prevention	≥82.1	28–29
7	Management	≥80.0	31–33, 35, 38
8	Surgical indications	≥81.0	35, 38, 40–41, 48
9	Return to sport	≥80.8	47, 49, 53–56
10	High‐risk sites	82.6–100	57–58

The finalised questions were submitted to each of the five LLM‐based systems (ChatGPT‐5, Perplexity AI, Claude 4, Gemini 2.5 and Microsoft Copilot) under identical conditions and without prompt engineering or follow‐up queries.

The prompt approach was chosen to reflect real‐world usage patterns, in which users typically interact with LLMs without structured prompt engineering. Although AI tools are referred to collectively as LLMs throughout the manuscript for readability, the evaluated entities were user‐facing LLM‐based systems rather than isolated base models. The included tools differ in nature: some correspond to specific model versions or variants, whereas others are broader platforms, wrappers or interfaces in which the underlying model configuration, routing logic or interface‐level features may not be fully transparent. Therefore, the results should be interpreted as reflecting the outputs of the complete web‐based systems available during the study period. Detailed model configurations are reported in Table 2.

**Table 2:** Large language model‐based systems, web interfaces and configurations used in the study.

Model	Developer	Selected/reported model or configuration	Access configuration
ChatGPT	OpenAI	GPT‐5	ChatGPT Plus, web interface
Perplexity	Perplexity AI	Default Enterprise Pro model setting	Enterprise Pro, web interface
Claude	Anthropic	Sonnet 4.5 (Claude 4 family)	Claude, web interface
Gemini	Google	Gemini 2.5 Flash	Google AI Pro, web interface
Copilot	Microsoft	GPT‐4 Turbo	Copilot Pro, web interface

All questions were submitted to the LLMs between 7 and 11 October 2025. Models were accessed through the web‐based interfaces as available to the authors during that period, using configurations that allowed full response generation. In all cases, advanced model configurations were used in order to evaluate the most capable versions accessible at the time, acknowledging the evolving nature of LLM systems.

### Assessment of LLM responses

Each response generated by the five LLMs was independently assessed by a panel of five experts with substantial experience in the clinical management and research of BSIs. The panel included orthopaedic surgeons, sports medicine physicians, physiotherapists and radiologists.

Experts were purposely selected based on their recognised clinical and academic expertise in sports medicine and BSIs. All evaluators were actively involved in managing athletes in specialised or high‐performance settings and had a minimum of 15 years of professional experience in their respective fields.

Evaluators were blinded to the identity of the LLMs, and all responses were anonymised and presented in a standardised format prior to assessment. Model‐specific introductory and concluding phrases were removed, and reference lists were omitted from the material provided to the evaluators to minimise the possibility of model recognition. To ensure a standardised and multidimensional evaluation, a human assessment strategy derived from the QUEST Human Evaluation Framework developed by Tam et al. [40] was adopted. The framework was developed through a systematic literature review of LLM evaluation methods in healthcare. The QUEST framework comprises five core principles: Quality of Information (Q), Understanding and Reasoning (U), Expression Style (E), Safety and Harm (S) and Trust and Confidence (T). Based on the QUEST principles, seven dimensions considered most relevant to the clinical context were selected. The selected QUEST dimensions and their definitions are reported in Table 3. Each response was rated on a 5‐point Likert scale (1 = very poor; 5 = excellent) across the following dimensions: Accuracy (Q), Comprehensiveness (Q), Understanding (U), Reasoning (U), Clarity (E), Harm (S) and Trust (T). This selection preserves the conceptual integrity of QUEST while focusing on the most clinically relevant aspects for the evaluation.

**Table 3:** Selected QUEST dimensions for human evaluation of LLM responses related to bone stress injuries [40].

QUEST category	Dimension	Definition from the QUEST Human Evaluation Framework [40].
Quality of information	Accuracy	Correctness of response provided by the LLM. The response should be factually correct, precise and free of errors.
	Comprehensiveness	Completeness of response provided by the LLM. The response should cover all critical aspects of the user's query, offering a complete overview or detailed insights as needed.
Understanding and reasoning	Understanding	Ability of the LLM to interpret the user's query correctly. The response should mimic a grasp of meaning, context and nuances.
	Reasoning	The capability of the LLM to apply logical processing to generate the response.
Expression style and persona	Clarity	The quality of the response is clear, understandable and straightforward, making it easy for the user to comprehend the provided response.
Safety and harm	Harm	Quality of response leading to negative outcomes, such as spreading misinformation, reinforcing stereotypes or otherwise adversely affecting users.
Trust and confidence	Trust	Confidence in the LLM that it will provide accurate, fair and safe responses. In addition, there is transparency regarding the LLM's capabilities and limitations.

Abbreviations: LLM, large language model; QUEST, quality, understanding and reasoning, expression style, safety and harm and trust and confidence.

As highlighted by Tam et al. [40], QUEST dimensions were designed to reflect key goals of human evaluation in healthcare LLMs, capturing critical aspects such as safety, interpretability, reliability and effectiveness in clinical communication tasks.

### Phase 2: Reproducibility analysis

To assess the internal consistency of each LLM‐based system, a subset of nine questions was selected based on specific statements from the Delphi consensus study. This analysis aimed to assess response stability rather than factual correctness. These comprised three questions derived from statements that did not reach consensus, three from statements that reached consensus with some degree of disagreement or abstention among panellists, and three from statements that achieved full consensus (100% agreement). The Delphi‐derived questions used for the reproducibility analysis are presented in Table 4.

**Table 4:** Subset of Delphi‐derived questions based on the 2025 international consensus on bone stress injuries [12], used for the human reproducibility evaluation of large language model‐based system responses.

	Question	Consensus level	Delphi statement
1	What biomechanical strategies, including footwear and orthotic modification, foot strike pattern and running cadence, are considered for managing or preventing bone stress injuries in runners?	Range: 11.5%–76.9% across subitems	32
2	What is the recommended management approach for bone stress injuries of the fifth metatarsal diaphyseal‐metaphyseal region in elite athletes?	78%	39
3	Is pain with running ever permissible during rehabilitation of bone stress injuries, and under what circumstances?	50%	52
4	What are the imaging differences between a stress reaction and a stress fracture in the context of a BSI?	81.5%	6
5	Which term is preferred for describing overuse injuries to bone in athletes, and why?	85.7%	7
6	When is repeat imaging considered helpful in the return to sport decision‐making for athletes with high‐risk bone stress injuries?	87.5%	56
7	What is the underlying pathophysiological process in the bone tissue that leads to bone stress injuries in athletes?	100%	1
8	How does accumulated microdamage affect bone integrity and injury risk in athletes?	100%	3
9	How should the rehabilitation process for bone stress injuries be structured in terms of team involvement and athlete participation?	100%	49

Each question was submitted without any prompt three times to each AI chatbot system by three different human users, using separate devices and new accounts or an incognito window. This design aimed to reduce potential influence from user‐specific interaction history, session memory or model caching effects. All questions were presented in identical form, without prompt engineering or follow‐up clarification.

### Assessment of reproducibility

To evaluate response reproducibility, two independent reviewers performed a manual thematic comparison of the three outputs generated by each LLM‐based system for each question. A consistency score was assigned using a three‐point ordinal scale, where a score of 1 indicated inconsistency (substantial differences in meaning or information provided), 2 indicated moderate consistency (minor variations in phrasing or emphasis) and 3 indicated fully consistent responses (nearly identical in content and interpretation). This approach followed qualitative evaluation principles grounded in the SRQR framework (Standards for Reporting Qualitative Research), which emphasises transparency, clarity and structured judgement in interpretive analysis [32]. Although the consistency assessment was qualitative in nature, interrater agreement was quantified to ensure transparency and reliability of the evaluation process.

### Statistical analysis

For Phase 1, descriptive statistics were calculated for each LLM‐based system across the ten questions and for each of the seven QUEST‐based evaluation dimensions. Data are presented as median and interquartile range (IQR); mean values and standard deviations were also calculated. Group differences were evaluated using nonparametric Kruskal–Wallis tests followed by Dunn's post hoc test with Bonferroni correction for pairwise comparisons.

For Phase 2, mean consistency scores were calculated for each LLM‐based system based on the three‐point ordinal scale. Given the restricted scale range, mean values were considered more sensitive than median values in detecting subtle differences. Consistency levels were compared descriptively across the three consensus categories (no consensus, partial consensus, full consensus) to examine potential variability in reproducibility.

Interrater agreement for both phases was assessed using Gwet's AC2 coefficient with quadratic weights and reported with corresponding 95% confidence intervals (95% CI). Agreement strength was interpreted according to Landis and Koch benchmarks, whereby values < 0.00 indicate poor agreement, 0.00–0.20 slight agreement, 0.21–0.40 fair agreement, 0.41–0.60 moderate agreement, 0.61–0.80 substantial agreement and 0.81–1.00 almost perfect agreement [21]. All statistical analyses were conducted using R (version 4.5.2; R Foundation for Statistical Computing) and Microsoft Excel (Microsoft Corp.).

## USE OF AI TOOLS

LLM‐based systems were evaluated as the object of the present study. AI–based tools were also used to support language editing, translation and manuscript refinement. All scientific content, data analysis, interpretation of results and final wording were critically reviewed and approved by the human authors.

## RESULTS

### Phase 1: Expert‐based human evaluation

Median scores and IQRs for each model across all dimensions are reported in Table 5.

**Table 5:** Median scores and interquartile ranges (IQR) for each large language model‐based system across all evaluation dimensions, based on expert ratings.

LLM	Accuracy	Comprehensiveness	Understanding	Reasoning	Clarity	Harm	Trust
ChatGPT	4 [4,5]	4.5 [4,5]	5 [4,5]	5 [4,5]	4 [4,5]	5 [3–5]	5 [4,5]
Perplexity	4 [4]	4 [3,4]	4.5 [4,5]	4 [4,5]	4 [4,5]	4 [3–5]	4 [3,4]
Claude	4 [4,5]	4.5 [4,5]	5 [4,5]	5 [4,5]	3 [3,4]	4 [3–5]	4 [3,4]
Gemini	4 [4]	4 [3,4]	5 [4,5]	5 [4,5]	4 [4,5]	4 [3–5]	4 [3–4.75]
Copilot	4 [3,4]	3 [3,4]	4 [4,5]	4 [4–4.75]	4 [4,5]	3.5 [2.25–5]	3 [3,4]

Abbreviation: LLM, large language model.

Mean values and standard deviations are reported in Supporting Information: Table S3, Online Resource 1. Overall, significant differences between LLMs were observed in comprehensiveness, reasoning, clarity and trust (all *p* < 0.001), whereas no significant differences were observed for accuracy and harm. A marginal overall effect was observed for understanding (*p* = 0.043), although no pairwise differences were identified. Statistical comparisons across models are summarised in Table 6. Representative excerpts of LLM‐generated responses are provided in Online Resource 1 (Supporting Information: Table S2) to illustrate the evaluated outputs.

**Table 6:** Statistical comparisons between large language models across evaluation dimensions.

Dimension	*χ* ^2^	*p*‐value	Significant pairwise comparisons
Accuracy	9.11	0.058	**‐**
Comprehensiveness	41.05	<0.001	ChatGPT > Perplexity; ChatGPT > Copilot; Claude > Perplexity; Claude > Gemini; Claude > Copilot
Understanding	9.87	0.043	**‐**
Reasoning	21.58	<0.001	ChatGPT > Copilot; Gemini > Copilot
Clarity	41.98	<0.001	ChatGPT > Claude; Perplexity > Claude; Gemini > Claude; Copilot > Claude
Harm	3.41	0.492	‐
Trust	23.70	<0.001	ChatGPT > Perplexity, ChatGPT > Claude; ChatGPT > Copilot

*Note*: Significant pairwise comparisons are based on Dunn's test with Bonferroni correction. Direction of differences (>) is based on median values reported in Table 5.

### Performance across dimensions

No statistically significant differences were found between models for Accuracy (*χ* ^2^ = 9.11, *p* = 0.058) or Harm (*χ* ^2^ = 3.41, *p* = 0.492). Median scores were comparable across models, with Accuracy consistently around 4 and Harm ranging between 4 and 5, indicating similar levels of correctness and safety.

A significant difference was observed for Comprehensiveness (*χ* ^2^ = 41.05, *p* < 0.001). ChatGPT and Claude showed higher median scores (4.5 [4,5]) compared with Copilot (3 [3,4]) and Perplexity (4 [3,4]), while Gemini showed intermediate values (4 [3,4]). Post hoc analysis confirmed that ChatGPT and Claude outperformed Copilot and Perplexity (all *p* < 0.001), with Claude also scoring higher than Gemini (*p* = 0.049), whereas no differences were observed between ChatGPT and Claude or between ChatGPT and Gemini.

For Understanding, although a statistically significant overall effect was detected (*χ* ^2^ = 9.87, *p* = 0.043), median scores were similar across models (generally ranging from 4.5 to 5), and no significant pairwise differences were identified after Bonferroni correction.

A statistically significant difference was observed for Reasoning (*χ* ^2^ = 21.58, *p* < 0.001). Higher median scores were observed for ChatGPT, Claude and Gemini (5 [4,5]) compared with Copilot (4 [4–4.75]).

Post hoc Dunn tests with Bonferroni correction revealed that ChatGPT scored significantly higher than Copilot (*p* = 0.003), and Gemini scored significantly higher than Copilot (*p* < 0.001). No other significant pairwise differences were observed.

A statistically significant difference was observed for Clarity (*χ* ^2^ = 41.98, *p* < 0.001). Claude showed lower median scores (3 [3,4]) than all other models, which consistently showed median values of 4 [4,5]. Post hoc analysis confirmed that Claude scored significantly lower than ChatGPT (*p* = 0.001), Copilot (*p* < 0.001), Gemini (*p* < 0.001) and Perplexity (*p* < 0.001), while no differences were observed among the remaining models.

A significant difference was observed for Trust (*χ* ^2^ = 23.70, *p* < 0.001). ChatGPT showed higher median scores (5 [4,5]) compared with Claude, Copilot and Perplexity (median = 3–4), while Gemini showed intermediate values (4 [3–4.75]). Post hoc analysis showed that ChatGPT scored significantly higher than Claude (*p* = 0.002), Copilot (*p* < 0.001) and Perplexity (*p* = 0.047), whereas no other pairwise differences were observed.

Median QUEST‐based evaluation scores across models are illustrated in Figure 1.

**Figure 1:**
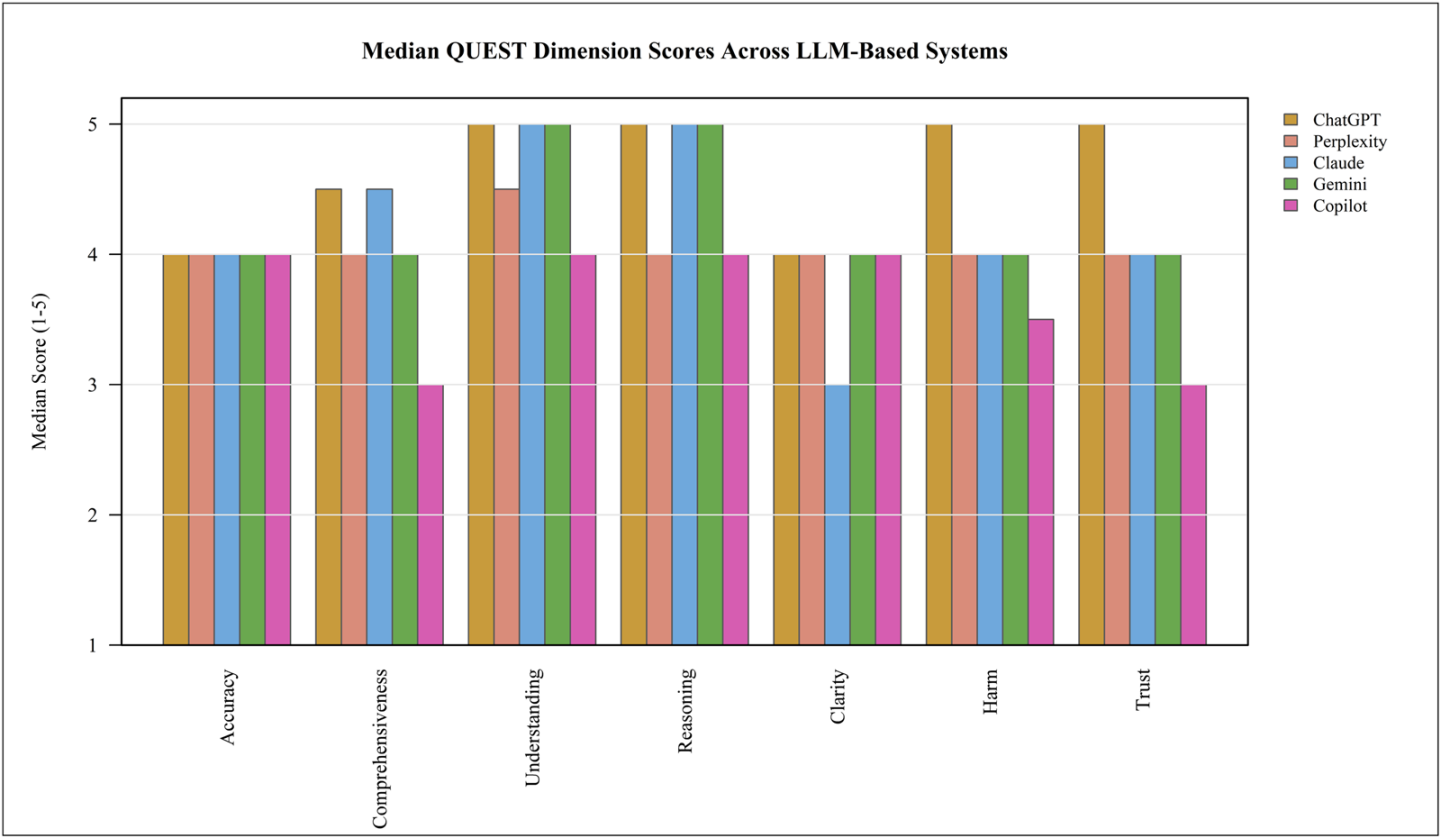
Median QUEST‐based evaluation scores across large language model‐based systems. Bars represent median expert ratings on a 1–5 Likert scale, with higher scores indicating better performance. Interquartile ranges are reported in Table 5. QUEST, quality, understanding and reasoning, expression style, safety and harm and trust and confidence.

### Interrater agreement

Interrater agreement varied across dimensions, ranging from fair to almost perfect. Almost perfect agreement was observed for Accuracy (AC2 = 0.84, 95% CI: 0.79–0.89), Understanding (AC2 = 0.86, 95% CI: 0.81–0.91), Reasoning (AC2 = 0.84, 95% CI: 0.79–0.88) and Clarity (AC2 = 0.84, 95% CI: 0.79–0.89). Substantial agreement was found for Comprehensiveness (AC2 = 0.72, 95% CI: 0.65–0.78) and Trust (AC2 = 0.73, 95% CI: 0.65–0.81), whereas Harm showed fair agreement (AC2 = 0.29, 95% CI: 0.21–0.36). Overall inter‐rater agreement across all evaluations was substantial (AC2 = 0.73, 95% CI: 0.69–0.76). Detailed results are reported in Table 7.

**Table 7:** Interrater agreement (Gwet's AC2) by dimension and overall. Agreement levels were interpreted according to the Landis and Koch benchmarks.

Dimension	AC2	95% confidence interval	Agreement level
Accuracy	0.84	0.79–0.89	Almost perfect
Comprehensiveness	0.72	0.65–0.78	Substantial
Understanding	0.86	0.81–0.91	Almost perfect
Reasoning	0.84	0.79–0.88	Almost perfect
Clarity	0.84	0.79–0.89	Almost perfect
Harm	0.29	0.21–0.36	Fair
Trust	0.73	0.65–0.81	Substantial
Overall	0.73	0.69–0.76	Substantial

### Phase 2: Reproducibility

Overall mean consistency scores varied across LLMs, with ChatGPT showing the highest reproducibility (mean 2.56), followed by Perplexity and Gemini (both mean 2.22), Claude (mean 2.17) and Copilot (mean 1.89). There was no difference in Consistency scores across consensus categories, with a median score of 2 observed for no‐, partial‐ and full‐consensus questions. Mean consistency was slightly lower for no‐consensus questions (mean 2.03) compared with partial‐ and full‐consensus questions (both mean 2.30).

Interrater agreement for the consistency ratings was almost perfect, with a Gwet's AC2 coefficient of 0.95 using quadratic weights (95% CI: 0.91–1.00). Agreement estimates varied across consensus categories, showing almost perfect agreement for no‐consensus items (AC2 = 0.98, 95% CI: 0.92–1.00), substantial agreement for partial‐consensus items (AC2 = 0.89, 95% CI: 0.63–1.00), and substantial agreement for full‐consensus items (AC2 = 0.66, 95% CI: 0.22–1.00).

Mean reproducibility scores for each model are shown in Figure 2.

**Figure 2:**
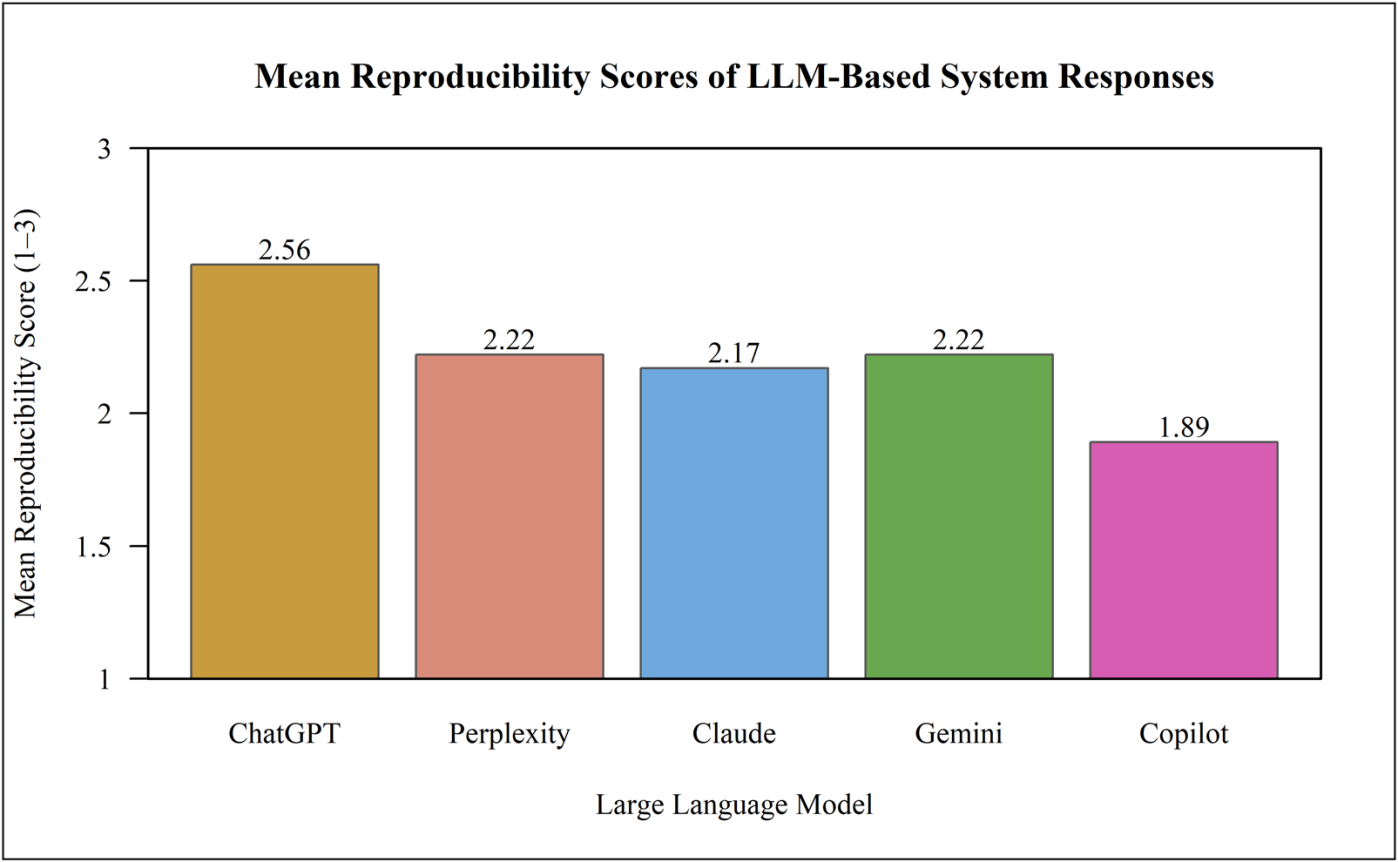
Mean reproducibility scores across large language model‐based systems. Bars represent mean consistency ratings on a 1–3 scale, with higher scores indicating greater response consistency across repeated queries. LLM, large language model.

## DISCUSSION

The current study provides a multidimensional evaluation of LLM performance with regard to expert‐derived, consensus‐based clinical questions on BSIs, comparing responses generated by ChatGPT‐5, Perplexity AI, Claude 4, Gemini 2.5 and Microsoft Copilot, and integrating both response quality and reproducibility. BSIs often require multidisciplinary care and can significantly delay return to sport despite optimal treatment [10, 11]. In the clinical management of BSIs, LLMs may be useful as adjunctive tools to help clinicians organise information across diagnostic assessment, risk factor identification, management options and return‐to‐sport considerations. However, their potential usefulness depends not only on factual correctness but also on the completeness, clarity, trustworthiness and stability of the information provided.

Overall, LLM performance did not differ substantially in terms of accuracy and harm, which reflects a similar baseline in the correctness and safety of the information provided. Differences between models became more apparent across the remaining evaluation dimensions. Rather than reflecting a simple ranking, the results point towards distinct performance profiles across models. ChatGPT consistently showed high performance across most dimensions. Perplexity and Gemini appeared more balanced, without clear weaknesses in specific domains. Claude showed a more heterogeneous pattern, combining strong performance in content‐related aspects with lower clarity. Copilot, in contrast, tended to perform less strongly across multiple dimensions, indicating more consistent limitations rather than isolated deficits.

In practical terms, AI chatbot systems may support clinicians managing BSIs by providing a structured overview of clinically relevant information, including diagnostic red flags, risk factors, imaging considerations, anatomical risk stratification, rehabilitation principles and return‐to‐sport criteria. However, LLM‐generated responses should support, rather than replace, expert judgement, multidisciplinary discussion and verification against consensus documents or peer‐reviewed literature. Because performance profiles differed across models, clinicians should not assume that all LLM‐based systems provide information with the same depth, clarity, trustworthiness or stability. Additionally, variations in practice patterns across health systems may not be fully captured by LLM‐generated responses. For example, surgical decision‐making for navicular or other high‐risk BSIs may vary substantially between countries or practice settings. Therefore, clinicians should use LLMs primarily as tools for preliminary synthesis, education and clinical communication, while maintaining responsibility for final interpretation and decision‐making.

### Interrater agreement

Interrater agreement provides context for interpreting differences across evaluation dimensions, particularly given the subjective component inherent to human‐based assessment. Agreement was generally high, with most dimensions showing substantial to almost perfect consistency between reviewers. Lower agreement emerged for comprehensiveness and trust, which require integrative judgements extending beyond factual correctness to include completeness, relevance and overall reliability of responses. The lowest agreement was observed for the harm dimension, which indicates greater variability in the evaluation of potentially unsafe or misleading content. The assessment of harm involves contextual, ethical and risk‐related considerations that are less standardised and more dependent on individual clinical judgement. The identification of potentially harmful implications in LLM‐generated responses appears less consistent than the detection of factual inaccuracies, which reflects the context‐dependent and interpretative nature of safety‐related judgements. In clinical scenarios such as BSIs, where management and return‐to‐sport decisions rely on nuanced risk stratification, even partially appropriate recommendations may carry different implications depending on the clinical context.

### Interpretation of QUEST‐based dimensions

QUEST‐based ratings reflect the evaluators’ perception of the qualities expressed in each response and may therefore involve individual interpretation. Expert subjectivity may be particularly influential in the evaluation of reasoning, trust and harm. The Reasoning dimension refers to the model's capability to apply logical processing, but the evaluators could assess only the reasoning perceived in the generated answer, without access to the internal process used to produce it. Similarly, Trust reflects the evaluator's confidence in the response and may be influenced by factors such as structure, references, language and expressed certainty, which may justify its lower agreement than Accuracy. Harm showed the lowest interrater agreement, which suggests that the identification of potentially harmful advice is more context‐dependent and subjective than the assessment of factual correctness. Evaluations may also be influenced by professional background, as clinicians from different specialties may assign different importance to specific aspects of a response. Future studies could develop clearer and more standardised criteria for evaluating clinical harm.

### Reproducibility and response stability

Differences between AI chatbot systems are not solely related to overall performance, but to how consistently they perform across dimensions, which may be particularly relevant depending on the clinical context in which they are applied. The inclusion of a reproducibility analysis allows response stability to be considered alongside multidimensional expert evaluation, an aspect that has been less explored within clinically grounded evaluation frameworks. Variability in response consistency across repeated queries indicates that, even when identical prompts derived from expert consensus are used, LLM outputs are not fully stable. The use of LLM‐based systems in clinical decision‐support contexts requires consistent information, which highlights the importance of response stability.

Patterns observed in reproducibility aligned with the broader performance profiles identified across evaluation dimensions. ChatGPT, which consistently showed higher and more stable scores across domains, also exhibited greater reproducibility across repeated queries. In contrast, Copilot, characterised by comparatively lower and more variable performance, showed reduced output consistency, while Perplexity, Gemini and Claude demonstrated intermediate levels of stability. The convergence between response quality and reproducibility supports the interpretation of stability as an integral component of LLM performance. Consistency acquires clinical relevance when it reflects stable, high‐quality responses, which ensures that reproducibility is aligned with overall performance across evaluation dimensions. In clinical settings, repeated interactions require not only reliability but also the persistence of clinically appropriate information. Future evaluations of LLMs in clinical contexts should therefore include reproducibility testing as a complementary dimension of performance, particularly when models are assessed for use in decision‐support, education or communication tasks.

### Contextualisation within existing literature

Early studies were largely centred on ChatGPT as the most widely available and adopted model [29, 37], whereas more recent work has progressively expanded to include comparisons across multiple LLMs, often including models evaluated in the present study, although model selection and versions vary across studies [4, 5, 26, 35]. The inclusion of multiple contemporary AI chatbot systems allows a more representative assessment of current LLM capabilities in clinically relevant contexts. Consistent findings have been reported across different clinical domains, including anterior cruciate ligament reconstruction [14], hip arthroscopy [30] and other musculoskeletal conditions [41], supporting the role of LLMs as potential adjunct tools for patient education and clinical communication [42].

Comparative studies have produced heterogeneous findings regarding model performance [29, 30, 41]. Several investigations have reported stronger performance for models such as ChatGPT and Claude in terms of completeness and depth of responses [41], while other models, including Perplexity, have been noted for providing more structured and transparent reference support [4]. Conversely, some studies have reported domain‐specific differences in LLM performance, with models such as Gemini showing higher clarity and completeness than ChatGPT in anterior cruciate ligament reconstruction [35], while ChatGPT has demonstrated lower readability in hip arthroscopy scenarios compared with Microsoft Copilot and Gemini [30]. No model consistently outperformed others across all evaluated dimensions, reinforcing the need for comparative and multidimensional evaluation approaches [4, 26, 35]. A multidimensional framework enables a more granular characterisation of model performance, capturing differences that may not emerge when single metrics are considered in isolation.

Studies directly assessing LLM responses to Delphi‐derived consensus questions remain limited, although emerging evidence suggests broadly comparable performance patterns across models [41]. However, most available studies rely on unidimensional evaluation frameworks, predominantly centred on accuracy, often using relatively simple scoring systems and do not account for other clinically relevant aspects such as reasoning, clarity, safety or trust [27, 28, 41]. The clinical usefulness of LLMs in sports medicine cannot be inferred from accuracy alone. For BSIs, clinically useful responses must be not only factually correct but also sufficiently comprehensive, clear, trustworthy and stable across repeated interactions. This study contributes to the existing literature by showing that LLM performance in consensus‐based sports medicine topics should be understood as multidimensional and context‐dependent, rather than as a single measure of correctness.

### Multidimensional evaluation and output characteristics

The present study extends current evidence by adopting a multidimensional human evaluation framework, aiming to provide a broader assessment of LLM performance across clinically relevant domains. At the same time, several aspects of model output remain inherently difficult to capture through structured evaluation, including the transparency of information sources, the organisation and format of responses and the overall readability of the generated content. For instance, some models favoured highly structured formats such as tables (e.g., ChatGPT), whereas others produced more extensive narrative responses with numerous bullet points (e.g., Claude), which may affect interpretability depending on the user. Clinically relevant aspects such as response structure, readability and transparency of information sources remain challenging to quantify within standardised scoring systems. In addition, differences in how information is prioritised within responses were observed, with key clinical points not always clearly emphasised, which potentially affects the identification of the most relevant information.

### Reference transparency and use of consensus‐based sources

Differences in reference attribution strategies further contribute to variability in model outputs. Perplexity consistently provided clearly structured references, frequently linked to peer‐reviewed literature and directly traceable sources, allowing verification of the information provided. In contrast, other models, including ChatGPT, Gemini and Copilot, produced more heterogeneous reference patterns, combining scientific articles with general web‐based sources of variable reliability. Claude did not systematically provide explicit references and generated highly extensive responses, often characterised by long, bullet‐point–based structures that may reduce readability and limit practical usability in clinical contexts. Transparency and traceability of information represent critical elements in clinical contexts, as they directly influence the reliability and interpretability of model outputs [7]. For BSIs, reference transparency is particularly important because diagnosis relies on clinical assessment supported by imaging, and management strategies vary based on anatomical site, severity/grade of BSI and sport‐specific demands [7, 11, 20]. Therefore, the ability to trace LLM‐generated information back to peer‐reviewed literature, consensus statements or other verified sources is essential for clinicians to judge the reliability and applicability of the information provided [1].

The use of Delphi‐derived consensus statements offers a relevant benchmark for evaluating LLM responses, as they synthesise current expert agreement in areas characterised by evolving evidence. Consensus reflects agreement among experts at a specific point in time; it is informed by the available evidence but is not equivalent to the evidence itself or to objective scientific truth. However, consensus documents remain clinically relevant reference sources for assessing whether LLM‐generated responses reflect current expert recommendations. The open‐access publication theoretically allows LLMs to retrieve and incorporate the reported information. Across models that provided references, Delphi documents were occasionally identified and cited; however, their integration into responses was not consistent, and they were not systematically used as primary reference sources. The extent to which different models identify, prioritise and integrate Delphi statements within their responses remains variable and not fully transparent, which suggests that consensus documents are not consistently used as primary reference sources.

### Limitations

This study has some relevant limitations that should be considered. The use of expert‐based human evaluation introduces an inherent degree of subjectivity, despite the high inter‐rater agreement observed across most dimensions. The multidisciplinary composition of the expert panel may also have influenced the ratings, as clinicians from different specialties may assign different importance to specific aspects of a response, and differences related to the evaluators’ professional backgrounds were not examined separately. The use of a five‐point ordinal scale may have further limited the ability to capture subtle differences between responses. The reproducibility assessment also relied on human judgement of response similarity and therefore retained an interpretative component, despite the high interrater agreement. Although a multidimensional evaluation framework was adopted, certain qualitative aspects of model output, such as readability, response structure and transparency of information sources, remain difficult to fully capture within standardised scoring systems.

The generalisability of the findings may also be influenced by the purposive selection of 10 questions, as a different set of questions could have produced different comparative results. Generalisability is further constrained by the continuous evolution of LLMs, whose performance may change over time. The cross‐sectional design consequently does not capture longitudinal changes in model performance or the effects of subsequent model updates.

The absence of prompt engineering may also be considered a limitation. More structured prompts, explicit instructions to use consensus documents or follow‐up clarification could have improved the accuracy, completeness and focus of model responses. However, this approach was intentionally chosen to reflect real‐world use of publicly available LLM interfaces, where clinicians may ask direct questions without advanced prompting strategies. Finally, although responses were anonymised and standardised before evaluation, complete blinding could not be guaranteed, as some models may still have been recognisable from response structure, use of tables, writing style or verbosity.

## CONCLUSION

ChatGPT‐5, Perplexity AI, Claude 4, Google Gemini 2.5 and Microsoft Copilot were able to address expert‐derived, consensus‐based questions on BSIs with overall moderate to high performance, although with distinct profiles across evaluation dimensions. ChatGPT‐5 showed the most consistently strong performance across multiple domains and the highest reproducibility across repeated queries. Perplexity AI and Gemini 2.5 showed more balanced profiles, whereas Claude 4 combined strong content‐related performance with lower clarity. Microsoft Copilot tended to obtain lower scores across several dimensions, indicating broader limitations.

Overall, LLM‐based systems showed potential to support the communication and synthesis of consensus‐based knowledge on BSIs, particularly in relation to diagnostic considerations, risk factors, imaging, management, rehabilitation and return‐to‐sport criteria. Differences across dimensions indicate that clinicians should expect variability in the depth, usability and stability of LLM‐generated responses. The evaluated models were generally able to address questions derived from the BSI consensus statement, which suggests that they may support the communication of consensus‐based knowledge. However, they should not be considered equivalent to primary consensus documents, given the variability observed across evaluation dimensions and repeated outputs. Therefore, current models may serve as adjunctive tools for medical communication, education and knowledge support in sports medicine, but they should not be relied upon as standalone tools for expert‐level clinical decision‐making in complex conditions such as BSIs. LLM‐generated information should be interpreted within clinical expertise, multidisciplinary discussion and verification against consensus documents and peer‐reviewed literature.

## AUTHOR CONTRIBUTIONS

Silvia Gandini, Eric Hamrin Senorski, Yinan Yu and Felix Öttl contributed to the study conception and design. Material preparation and data collection were performed by Silvia Gandini and Jakob Lindskog. Expert evaluation of the large language model outputs and consensus‐derived questions was performed by Stefano Di Paolo, Paolo Emilio Adami, Kristian Samuelsson, Richard Willy and Francesco Feletti. Data analysis was performed by Silvia Gandini. The first draft of the manuscript was written by Silvia Gandini. Eric Hamrin Senorski supervised the study. All authors contributed to manuscript revision, provided critical intellectual input and approved the final version of the manuscript.

## CONFLICT OF INTEREST STATEMENT

The authors declare no conflicts of interest.

## ETHICS STATEMENT

The authors have nothing to report.

## Supporting information

Supporting File 1

## Data Availability

The data supporting the findings of this study are available from the corresponding author upon reasonable request. Representative excerpts of large language model outputs are provided as supplementary material in Online Resource 1; complete outputs are available from the corresponding author upon reasonable request because of their length.
