# Supplementary material for: Large language models show distinct multidimensional performance profiles and variable reproducibility in consensus‐based bone stress injury
Source: J Exp Orthop. 2026 Oct 7;13(4):e70939. doi: 10.1002/jeo2.70939 (PMC13644707; doi:10.1002/jeo2.70939)
Supplement: Supplementary file 1 [file JEO2-13-e70939-s001.docx]

**Online Resource 1**
**Supplementary material for:**
**Large Language Models Show Distinct Multidimensional Performance Profiles and Variable Reproducibility in Consensus-Based Bone Stress Injury Questions**

| *Table S1* *Delphi-based questions used to query LLMs.* | | | | |
| --- | --- | --- | --- | --- |
|  | **Clinical domain** | **Consensus level** | **Statement** | **Question** |
| 1 | Pathophysiology | ≥96.4% | 1, 2, 3, 4 | What is the pathophysiological mechanism underlying bone stress injuries, and why are they considered overuse injuries that develop along a pathological continuum? |
| 2 | Terminology & classification | ≥81.5% | 5, 6, 7, 8, 13, 14 | What terminology and classification systems are recommended for bone stress injuries in athletes, and how do they support accurate diagnosis, treatment planning, and clinical communication, while also considering the predominant bone composition (trabecular-rich vs. cortical-rich sites) at the location of the injury? |
| 3 | Clinical suspicion | Range 79.3–96.6% across sub-items | 15 | What are the key etiological elements and clinical signs that should raise suspicion for a bone stress injury in an athlete? |
| 4 | Diagnostic assessment | ≥82.1% | 17, 18, 19, 20, 21, 24, 26, 31 | What imaging, clinical, and laboratory assessments are recommended for diagnosing bone stress injuries and evaluating bone health in athletes, and how should each be applied in clinical practice? |
| 5 | Risk factors | Range 88.0%–100% across sub-items | 23 | What are the main intrinsic and extrinsic risk factors for developing a bone stress injury in athletes, and how do these differ across populations such as runners, dance/performing arts, military personnel, and court/field/jumping athletes? |
| 6 | Prevention | ≥82.1% | 28, 29 | What physical activity strategies are recommended to promote bone health and reduce the risk of bone stress injuries in athletes, particularly during growth and development? |
| 7 | Management | ≥80.0% | 31, 32, 33, 35, 38 | How are bone stress injuries managed in athletes, and what factors influence treatment decisions? |
| 8 | Surgical indications | ≥81.0% | 35, 38, 40, 41, 48 | What factors support surgical treatment of bone stress injuries, and how should fifth metatarsal stress fractures be managed in elite athletes? Does surgical management of navicular stress injuries differ according to grade or classification? |
| 9 | Return to sport | ≥80.8% | 47, 49, 53, 54, 55, 56 | What clinical criteria and team-based considerations are essential to guide progression to running and return to sport after a bone stress injury? |
| 10 | High-risk sites | Range 82.6–100% across sub-items | 57, 58 | Which anatomical sites are considered high-risk due to their potential for complications and may require surgical management, and which sites are typically managed non-surgically despite the presence of a stress fracture? |

| *Table S2 Representative excerpts of responses generated by large language models for a representative question. Responses are presented as verbatim excerpts from model outputs, with omissions indicated by brackets […] and no modifications to original wording.* | |
| --- | --- |
| **Question: What clinical criteria and team-based considerations are essential to guide progression to running and return to sport after a bone stress injury?** | |
| **Model** | **Response (excerpt)** |
| **ChatGPT-5** | “Return-to-sport (RTS) after a BSI should be progressive, individualized, and multidisciplinary, emphasizing both biological healing and functional readiness. […] Athletes must meet all of the following clinical readiness criteria […] Pain-free at rest […] No local tenderness on firm palpation […] single-leg hop ×10 without pain […] A coordinated multidisciplinary team […] is essential for safe progression.” |
| **Perplexity AI** | “Progression to running and return to sport […] should be guided by specific clinical criteria […] Resolution of bone tenderness […] Pain-free walking […] Radiological healing (high-risk sites) […] Strength, functional, and loading tests […] A multidisciplinary team approach is essential […] with gradual, individualized progression.” |
| **Claude 4** | “Return to sport requires a systematic, multi-phase approach with objective clinical criteria, comprehensive team involvement […] Key criteria include […] complete pain-free status […] no bone tenderness to palpation […] strength, flexibility, and endurance restored […] A multidisciplinary team […] ensures all determinants of healing are addressed.” |
| **Gemini 2.5** | “The decision should never be based on time alone […] the athlete must be completely pain-free during all activities of daily living […] The ability to perform 10–15 pain-free hops […] is a strong indicator […] A successful return to sport is a collaborative process involving a multidisciplinary team […] to prevent re-injury.” |
| **Microsoft Copilot** | “Progressing to running and returning to sport […] requires a structured, criteria-based approach […] Pain-free at rest and during daily activities […] Pass single-leg hop test without pain […] Imaging […] especially important for navicular, femoral neck […] A multidisciplinary […] team […] should guide progression and ensure safe return.” |

| *Table S3 Mean scores for each large language model across all evaluation dimensions. Values are presented as mean ± standard deviation (SD), calculated across all questions and raters for each evaluation dimension.* | | | | | | | |
| --- | --- | --- | --- | --- | --- | --- | --- |
| **LLM** | **Accuracy** | **Comprehensiveness** | **Understanding** | **Reasoning** | **Clarity** | **Harm** | **Trust** |
| **ChatGPT-5** | 4.10 ± 0.79 | 4.34 ± 0.77 | 4.56 ± 0.67 | 4.52 ± 0.74 | 4.12 ± 0.80 | 3.90 ± 1.30 | 4.24 ± 0.96 |
| **Perplexity AI** | 4.04 ± 0.67 | 3.54 ± 1.05 | 4.26 ± 0.92 | 4.24 ± 0.82 | 4.32 ± 0.62 | 3.82 ± 1.32 | 3.80 ± 0.64 |
| **Claude 4** | 4.04 ± 0.88 | 4.38 ± 0.78 | 4.48 ± 0.81 | 4.34 ± 0.85 | 3.56 ± 0.70 | 3.78 ± 1.15 | 3.62 ± 0.85 |
| **Gemini 2.5** | 4.08 ± 0.63 | 3.94 ± 0.77 | 4.66 ± 0.52 | 4.64 ± 0.48 | 4.46 ± 0.54 | 3.72 ± 1.34 | 3.88 ± 0.92 |
| **Copilot** | 3.76 ± 0.62 | 3.56 ± 0.86 | 4.38 ± 0.53 | 4.06 ± 0.68 | 4.18 ± 0.83 | 3.50 ± 1.25 | 3.48 ± 0.93 |
